# The dual functions of KDM7A in HBV replication and immune microenvironment

**DOI:** 10.1128/spectrum.01641-23

**Published:** 2023-08-25

**Authors:** Di Yang, Renyun Tian, Rilin Deng, Binbin Xue, Shun Liu, Luoling Wang, Huiyi Li, Qian Liu, Mengyu Wan, Songqing Tang, Xiaohong Wang, Haizhen Zhu

**Affiliations:** 1 Institute of Pathogen Biology and Immunology of College of Biology, Hunan Provincial Key Laboratory of Medical Virology, State Key Laboratory of Chemo/Biosensing and Chemometrics, Hunan University, Changsha, Hunan, China; 2 Key Laboratory of Tropical Translational Medicine of Ministry of Education, Department of Pathogen Biology and Immunology, Institute of Pathogen Biology and Immunology, School of Basic Medicine and Life Science, The University of Hong Kong Joint Laboratory of Tropical Infectious Diseases, The First Affiliated Hospital and The Second Affiliated Hospital of Hainan Medical University, Hainan Medical University, Hainan, China; Institute of Microbiology Chinese Academy of Sciences, Beijing, China

**Keywords:** KDM7A, HBV, IFN-γ, JAK/STAT signaling pathway, macrophages

## Abstract

KDM7A (lysine demethylase 7A, also known as JHDM1D) is a histone demethylase, it is mainly involved in the intracellular post-translational modifications process. Recently, it has been proved that the histone demethylase members can regulate the replication of hepatitis B virus (HBV) and the expression of key molecules in the Janus-activated kinase-signal transducer and activator of the transcription (JAK/STAT) signaling pathway by chromatin modifying mechanisms. In our study, we identify novel roles of KDM7A in HBV replication and immune microenvironment through two subjects: pathogen and host. On the one hand, KDM7A is highly expressed in HBV-infected cells and promotes HBV replication *in vitro* and *in vivo*. Moreover, KDM7A interacts with HBV covalently closed circular DNA and augments the activity of the HBV core promoter. On the other hand, KDM7A can remodel the immune microenvironment. It inhibits the expression of interferon-stimulated genes (ISGs) through the IFN-γ/JAK2/STAT1 signaling pathway in both hepatocytes and macrophages. Further study shows that KDM7A interacts with JAK2 and STAT1 and affects their methylation. In general, we demonstrate the dual functions of KDM7A in HBV replication and immune microenvironment, and then we propose a new therapeutic target for HBV infection and immunotherapy.

**IMPORTANCE:**

Histone lysine demethylase KDM7A can interact with covalently closed circular DNA and promote the replication of hepatitis B virus (HBV). The IFN-γ/JAK2/STAT1 signaling pathway in macrophages and hepatocytes is also downregulated by KDM7A. This study provides new insights into the mechanism of HBV infection and the remodeling of the immune microenvironment.

## INTRODUCTION

Hepatitis B virus (HBV) belongs to a hepatophilic DNA envelope virus with a partially double-stranded, relaxed circular DNA (rcDNA) genome of ~3.2 kb containing four partially overlapping open reading frames (ORFs), namely, P (polymerase), S (surface), C (core), and X (HBx protein) (1). HBV serves as a key factor in the development of chronic hepatitis, cirrhosis, and even liver cancer. Approximately, 257 million people worldwide have been infected with HBV (data from WHO 2017). Following HBV entry into the cell, the rcDNA genome is translocated into the nucleus and converted into covalently closed circular DNA (cccDNA) using host cell DNA repair reactions. The covalently closed circular DNA provides the only template for viral transcription and can form a stable minichromosome in the nuclei of infected hepatocytes. The stable minichromosome consists of cccDNA, histone 3 (H3), H4 proteins, non-histone proteins such as hepatitis B core protein (HBc) and hepatitis B x protein (HBx), and host transcription factors (2). The difficulty of eradicating HBV-derived cccDNA in patients leads to the failure of viral clearance, and the inability of the immune system to mount effective immune responses against HBV can also contribute to it (3). There are two common ways to treat HBV in the clinic: direct inhibition of HBV replication by nucleoside or nucleotide analogs (NUCs) and modulation of immune responses by interferon (IFN) (1).

Interferon is a key regulator of the immune response process against viruses, and it is also one of the first lines of defense for host cells against viruses (4). IFNs play myriad roles in innate and adaptive immunity, and there are three types of IFN: I (α, β, ω, ε, and κ), II (γ), and III (λ). In the human body, type I and type III interferons act primarily as antiviral agents (5), while type II interferon (IFN-γ) mainly participates in tissue homeostasis, immune and inflammatory responses, and tumor immune monitoring. IFN-γ, originally identified as a “macrophage activating factor,” is primarily produced by immune system cells, including innate-like lymphocyte populations and adaptive immune cells (6). IFNs can activate the Janus-activated kinase (JAK)-signal transducer and activator of the transcription (STAT) pathway. The JAK/STAT signaling pathway represents a ubiquitously expressed intracellular signaling pathway that participates in many important biological processes, including cell proliferation, differentiation, and immune regulation (7). It is composed of ligand-receptor complexes, JAKs, and STATs. There are four members in the JAK family: JAK1, JAK2, JAK3, and TYK2. The STAT family comprises seven members: STAT1, STAT2, STAT3, STAT4, STAT5a, STAT5b, and STAT6 (8). Our current study mainly focuses on the JAK2/STAT1 signaling pathway activated by IFN-γ in hepatocytes and macrophages.

Lysine demethylase 7A (KDM7A, also known as JHDM1D) is a histone demethylase that is mainly involved in intracellular post-translational modification (PTM). It consists of plant homeodomain (PHD), finger protein (PHF), and Jumonji (Jmj) domains. KDM7A is a dual histone demethylase that functions as a transcription activator, whose common targets are lysine 9 of histone H3 (H3K9me1/2) and lysine 27 of histone H3 (H3K27me1/2) (9). Reportedly, KDM7A plays a role in the development of cancers (10 – 13), neural differentiation (14), and inflammatory responses (15). Moreover, it can affect the development of hepatic steatosis (16). As a member of a homologous family, KDM3A functions as an essential factor for the activation of the JAK2-STAT3 signaling pathway (17). However, the regulation of KDM7A in the JAK-STAT signaling pathway has not been reported.

At present, HBV is usually described in two words: cunning and stealthy. It is “cunning” because it results in the stability of cccDNA and “stealthy” is due to the inability of the immune system to mount effective immune responses against HBV. As the most important component of HBV, cccDNA is assembled with cellular histone proteins into chromatin. Furthermore, a previous study has shown that cccDNA chromatin has high levels of active post-translational modifications enriched at specific regions of the HBV genome (18), and methyltransferase inhibits the transcription of cccDNA by increasing methylation of cccDNA-involved histones (19). However, cccDNA modified by KDM7A has not been elucidated. In this study, we identified new roles of KDM7A through two subjects: pathogen and host. One role is to promote HBV replication by interacting with cccDNA and facilitating the activation of the HBV core promoter. The other is to remodel the immune microenvironment. KDM7A inhibits the expression of interferon-stimulated genes (ISGs) through the IFN-γ/JAK2/STAT1 signaling pathway in both hepatocytes and macrophages.

## RESULTS

### KDM7A is highly expressed in HBV-infected cells

**Fig 1:**
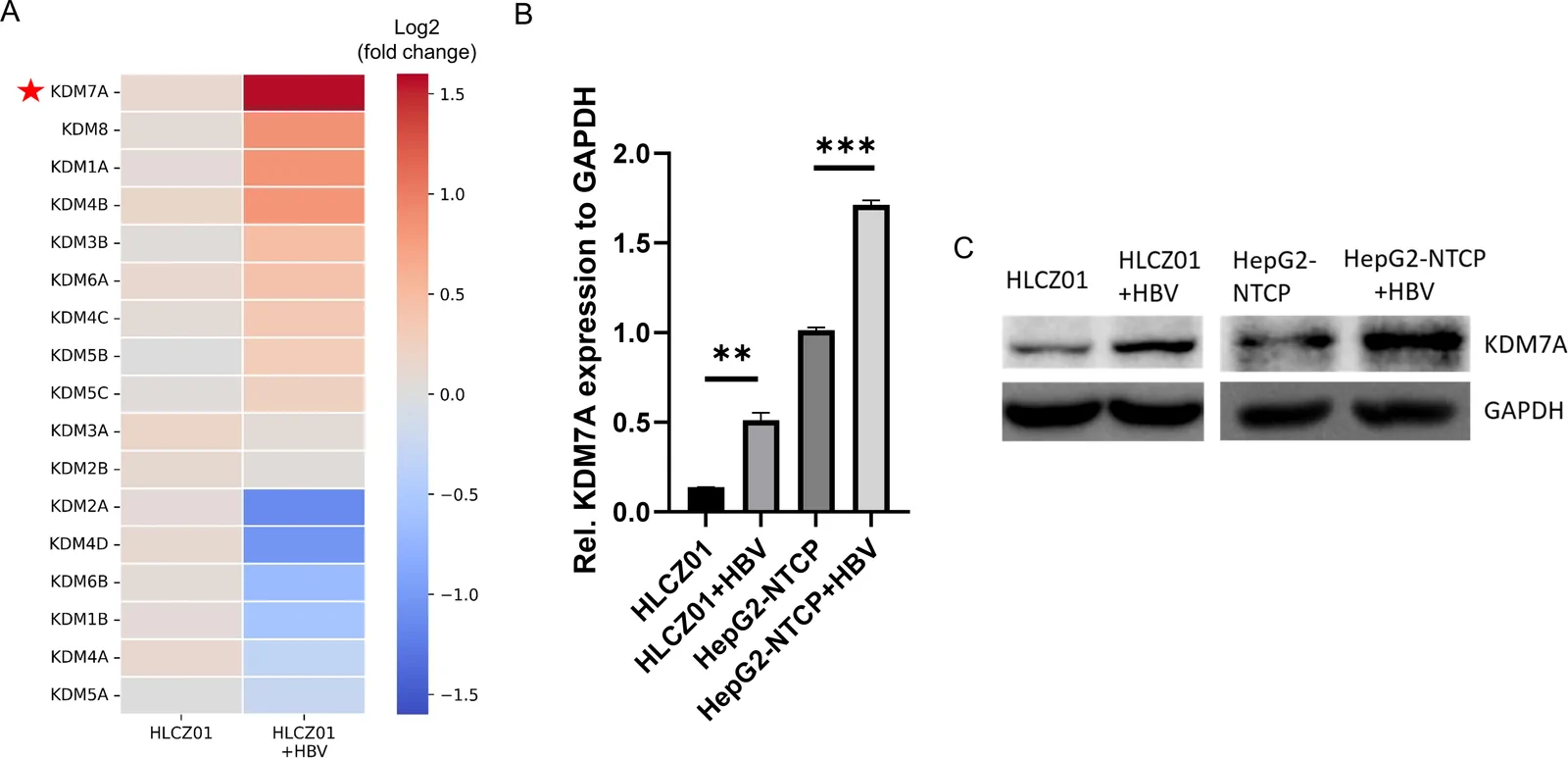
KDM7A is highly expressed in HBV-infected cells. (**A**) Heatmap of changes in the mRNA expression level of KDM family members in HBV-infected HLCZ01 cells. Log2 (fold change) values were calculated from fragments per kilobase of transcript per million values of RNA-seq. (**B**) qPCR analysis of KDM7A mRNA expression in HLCZ01/HBV-infected HLCZ01 cell lines and HepG2-NTCP/HBV-infected HepG2-NTCP cell lines and normalized with GAPDH. (**C**) Western blot analysis of the protein expression level of KDM7A in HLCZ01/HBV-infected HLCZ01 cell lines and HepG2-NTCP/HBV-infected HepG2-NTCP cell lines. Experiments were independently repeated two or three times with similar results. The results are means and standard deviations. **, *P*＜0.01; ***, *P*＜0.001 (versus control).

Previous studies have shown that cccDNA chromatin has high levels of active post-translational modifications (18), and methyltransferase inhibits the transcription of cccDNA by increasing the methylation of cccDNA-bound histones (19). However, the effect of histone demethylases on the regulation of HBV cccDNA function remains obscure. As a demethylase related to PTMs, we wondered whether the KDM family affects the replication of HBV; thus, we first measured the expression of the KDM family by RNA sequencing (RNA-seq). HLCZ01 is a new type of human liver cancer cell line constructed in our laboratory that presents the complete life cycle of hepatitis B virus and hepatitis C virus (HCV) (20). The RNA analysis showed that by comparing KDM7A to some of its family members, the expression difference of KDM7A was the most significant between the experimental group (HBV-infected HLCZ01) and the control group (HLCZ01) (Fig. 1A). Sodium taurocholate cotransporting polypeptide (NTCP) is a multiple transmembrane transporter predominantly expressed in the liver and is essential to HBV infections (21). A HepG2 cell line with stable expression of NTCP was screened and infected with HBV in our study. To further confirm the results, we tested the relative mRNA level of KDM7A in HepG2-NTCP/HBV-infected HepG2-NTCP cells and HLCZ01/HBV-infected HLCZ01 cells by qRT-PCR. KDM7A was indeed highly expressed in HBV-infected hepatocytes (Fig. 1B). Furthermore, the same results were also observed at the protein level by western blotting (Fig. 1C). Overall, these data demonstrated that HBV infection promoted the expression of KDM7A, implying that KDM7A might be involved in regulating HBV replication.

### KDM7A promotes HBV replication *in vitro*

**Fig 2:**
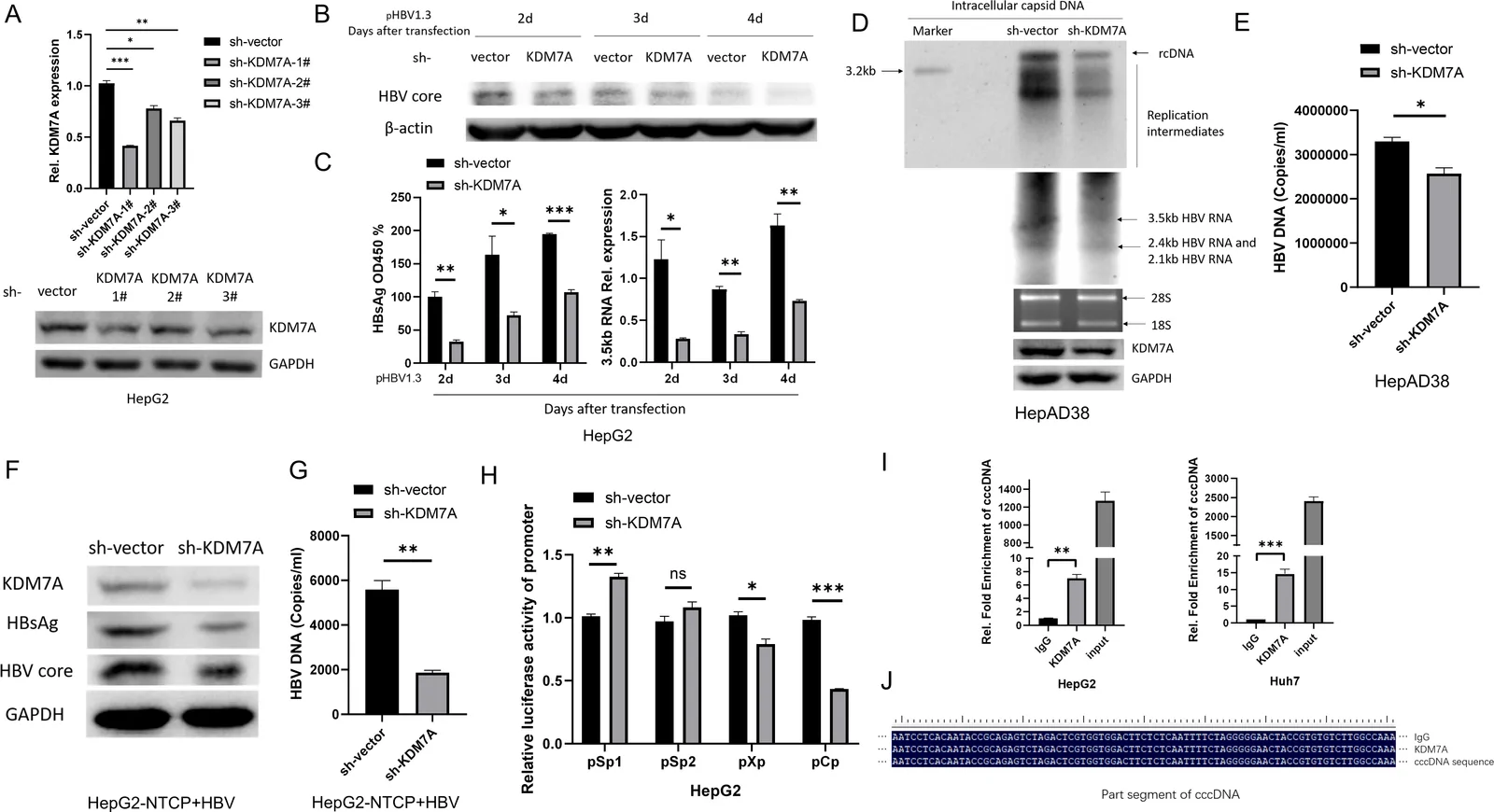
KDM7A promotes HBV replication *in vitro*. (**A**) Three different sequences of lentiviral plasmids targeting KDM7A and the control group were designed to infect HepG2 cells, the infected cells were selected with puromycin (2 ug/mL) to screen the best growing single cell clones for their ability to knock down KDM7A expression. Western blot analysis and qPCR analysis of KDM7A in these four cell lines normalized with GAPDH. (**B**) HepG2-sh-vector and HepG2-sh-KDM7A cell lines were transfected with pHBV1.3. Western blot analysis of HBV core protein (**B**), ELISA of HBsAg and qRT-PCR of 3.5 kb HBV RNA normalized with GAPDH (**C**) at 2, 3, and 4 days after transfection was performed. HepAD38 cells were infected with lentivirus harboring sh-vector/KDM7A respectively for 72 h. Southern blot analysis of intracellular capsid DNA of HBV, northern blot analysis of HBV RNA, and western blot analysis of KDM7A were performed (**D**). The viral DNA in the supernatant as determined by real-time PCR was shown as the number of HBV copies per milliliter of supernatant (**E**). HepG2-NTCP cells were infected with lentivirus harboring sh-vector/KDM7A, respectively, for 72 h, and then HepG2-NTCP cells were infected with HBV for 9 days. Western blot analysis of HBsAg, HBV core, and KDM7A was shown (**F**). The viral DNA in the supernatant as determined by real-time PCR was shown as the number of HBV copies per milliliter of supernatant (**G**). (**H**) HepG2-sh-vector cells and HepG2-sh-KDM7A cells were transfected with pSP1-Luc, pSP2-Luc, pXp-Luc, or pCp-Luc, respectively. Luciferase activity analysis of pSP1, pSP2, pXp, and pCp. (**I**) HepG2 and Huh7 were transfected with prcccDNA and Cre recombinase. Chromatin immunoprecipitation (ChIP) analysis of the relative fold enrichment of cccDNA in IgG, KDM7A, and input was performed. GAPDH was used as the internal control. (J) The binding sequences of IgG and KDM7A and part segment of cccDNA were performed. Experiments were independently repeated two or three times with similar results. The results are means and standard deviations. ns, no significance; *, *P*＜0.05; **, *P*＜0.01; and ***, *P*＜0.001 (versus control).

Therefore, we next focused on investigating the role of KDM7A in HBV infection. To expound the effect of KDM7A on HBV replication, our experiments were conducted in three types of cell lines related to HBV: HepG2, HepAD38, and HepG2-NTCP cell lines. In addition, we constructed two kinds of lentivirus-packaged short hairpin RNAs, one of which was a blank control, and the other three were designed to target KDM7A. After the expression of KDM7A was stably inhibited in HepG2 cells by lentivirus harboring short hairpin RNA (shRNA) (Fig. 2A), we transfected pHBV1.3, which can simulate the genome of HBV, into HepG2 cells. The expression of the HBV core protein was tested on the second, third, and fourth days after pHBV1.3 transfection, and we found that KDM7A knockdown decreased the HBV core protein level (Fig. 2B). Furthermore, the optical density of HBsAg by ELISA and the relative expression of 3.5 kb HBV RNA by qRT-PCR showed the same trend (Fig. 2C). Similarly, we detected other indices related to HBV in the HepAD38 cell line, which can express HBV under the control of the inducible tetracycline promoter (22). The HBV DNA and HBV RNA in HepAD38 cells were collected after the cells were successfully infected with lentivirus harboring shRNA, and the intracellular capsid DNA of HBV and the transcripts of HBV were obviously reduced due to the low expression of KDM7A shown by Southern blot and Northern blot (Fig. 2D). The quantity of HBV DNA showed the same result by qPCR (Fig. 2E). For a more comprehensive validation, following infection of HBV-infected HepG2-NTCP cells with lentivirus harboring shRNA, we detected the expression of HBsAg and HBV core by western blot (Fig. 2F) and the quantity of HBV DNA by qPCR (Fig. 2G). All the data demonstrated that the low expression of KDM7A can inhibit HBV replication. KDM7A can bind to promoters and function as an activator to affect the stability of genes (13). We speculated that KDM7A may bind to HBV promoters and thus affect HBV replication. Translation of HBV is regulated by four promoters, namely, the core antigen (HbcAg) promoter (Cp), surface antigen promoters 1 and 2 (SP1 and SP2), and X protein (HBx) promoter. These four promoters can produce 3.5, 2.4, 2.1, and 0.7 kb HBV RNA products, respectively (23). A dual-luciferase reporter assay in HepG2 cells showed that KDM7A affected SP1, XP, and Cp. Moreover, the effect of KDM7A on Cp was the most obvious. The knockdown of KDM7A decreased Cp promoter activity (Fig. 2H). HBV cccDNA, the only template in viral transcription, can form a stable minichromosome in the nuclei of infected hepatocytes. The longevity of cccDNA is believed to be a key factor in HBV chronicity. It is important to clarify the factors affecting the presence of cccDNA in hepatocytes (3). To test whether KDM7A interacts with cccDNA, the recombinant cccDNA (rcccDNA) system was used in this study. In this system, the transfected precursor rcccDNA (prcccDNA) can be modified by Cre recombinase to form rcccDNA, which is artificially inserted in a small loxP-chimeric intron compared to HBV-encoded cccDNA. After Cre/loxp-mediated DNA recombination, the intron of rcccDNA will be removed post-transcriptionally through splicing so that it can be used as a marker of cccDNA (24). As shown in Fig. 2I, the enrichment of cccDNA on KDM7A was higher than that in the control in HepG2 and Huh7 cells. Subsequently, we compared the DNA sequences enriched on the KDM7A protein in both cell lines with the cccDNA sequences. We found that the target sequences were consistent with the cccDNA sequences (Fig. 2J). Collectively, these data demonstrated that KDM7A might promote HBV replication by interacting with cccDNA and augmenting the activity of the HBV core promoter.

### KDM7A promotes HBV replication *in vivo*

**Fig 3:**
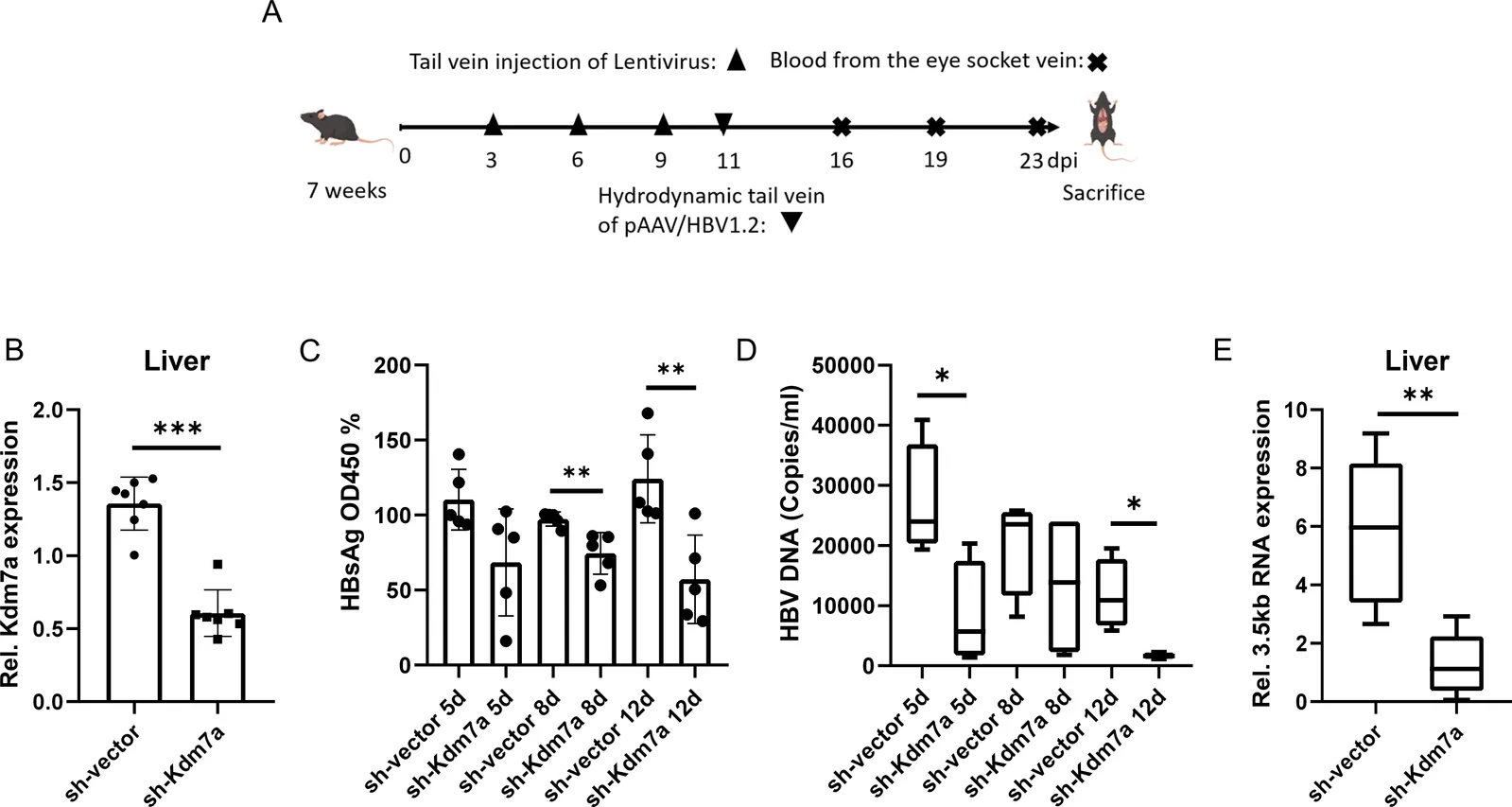
KDM7A augments HBV replication *in vivo*. (**A**) Lentivirus harboring sh-vector or sh-Kdm7a was injected into mice at 7 weeks of age, once every 3 days, three times in total. Two days after the injection of lentivirus, pAAV/HBV1.2 were injected via HTV (hydrodynamic tail vein) injection. Blood was collected from the eye socket vein over the next 5, 8, and 12 days. Mice were executed after anesthesia and subsequently dissected to obtain liver tissue. (**B**) Mouse liver RNA was extracted, and qPCR analysis of Kdm7a was performed. GAPDH was used as the internal control. Valid data (*n* = 7). ELISA analysis of HBsAg (**C**) and qPCR of HBV DNA (**D**) from mice serum were detected at 5, 8, and 12 days after HTV of pAAV/HBV1.2. The calculation method of HBV DNA was mentioned above. Valid data (*n* = 5). (**E**) Mouse liver RNA was extracted, and qPCR analysis of 3.5 kb RNA was performed. GAPDH was used as the internal control. Valid data (*n* = 5). Experiments were independently repeated two or three times with similar results. The results are means and standard deviations. *, *P*＜0.05; **, *P*＜0.01; ***, and *P*＜0.001 (versus control).

To investigate the physiological function of KDM7A in HBV replication, we constructed a C57BL/6 mouse model with a knockdown of Kdm7a. First, we knocked down Kdm7a expression in mice via intravenous injection of shRNA lentivirus, which can effectively reduce the expression of target genes in mice (25). The intravenous injection of lentivirus harboring shRNA was conducted three times in 9 days. Subsequently, pAAV/HBV1.2 with long-term HBV infection was injected into mice by hydrodynamic tail vein (HTV) injection 2 days later (26). Blood was collected from the eye socket vein on days 5, 8, and 12 (Fig. 3A). The silencing effect on KDM7A mRNA levels in mice is shown in Fig. 3B. Consistent with the *in vitro* data, the knockdown of Kdm7a reduced the level of HBsAg in blood serum at 8 and 12 days (Fig. 3C). Moreover, we compared the quantity of HBV DNA in mouse serum and the relative HBV 3.5 kb RNA level in mouse liver by qPCR between the experimental and control groups. Knockdown of Kdm7a decreased HBV DNA at 5 and 12 days and decreased HBV 3.5 kb RNA level after HTV injection of pAAV/HBV1.2 (Fig. 3D and E). All the data supported that KDM7A promotes HBV replication *in vivo*.

### HBV promotes the expression of KDM7A in macrophages by direct exposure

**Fig 4:**
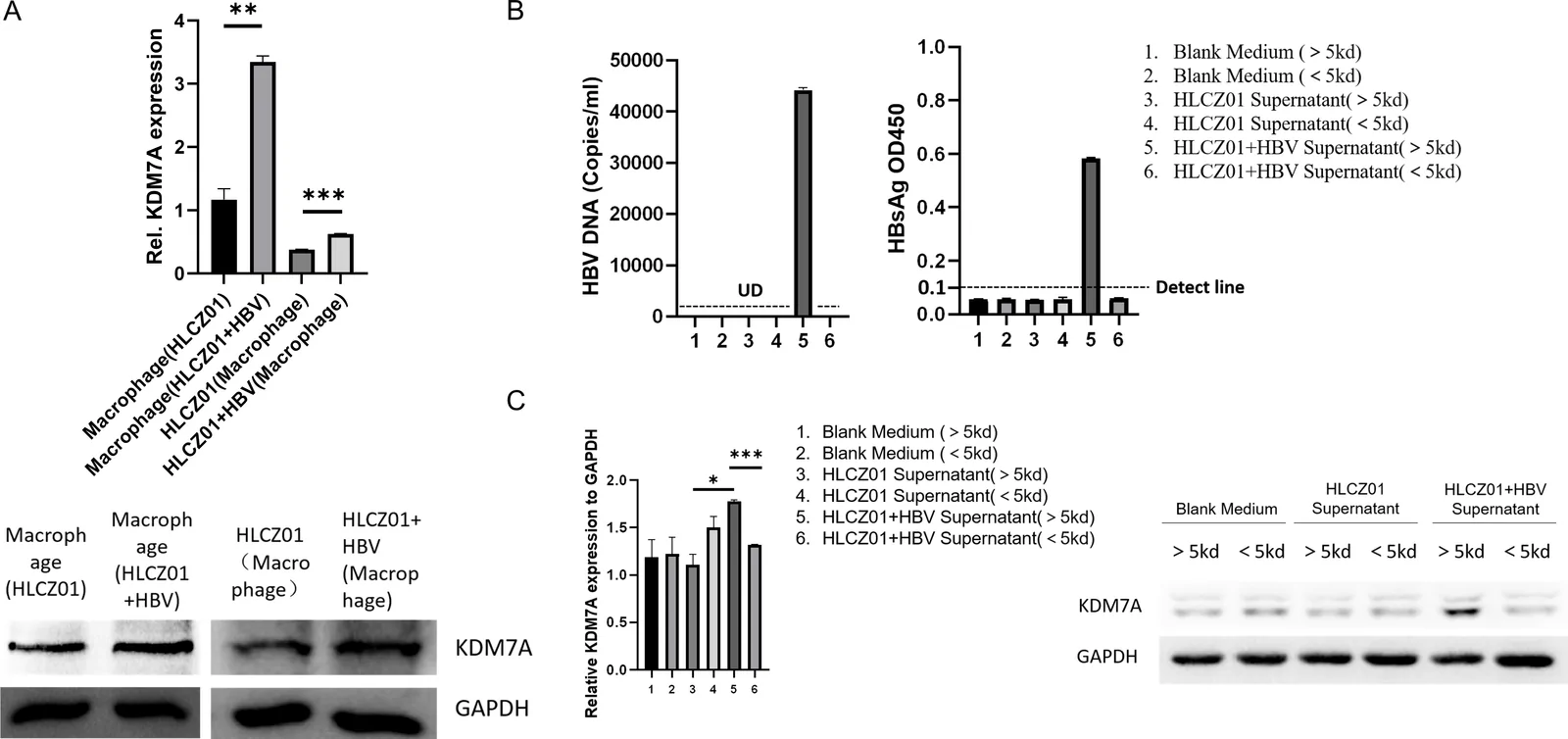
HBV promotes the expression of KDM7A in macrophages by direct exposure. (**A**) qPCR analysis and western blot analysis of KDM7A in coculturing cells. Macrophage (HLCZ01)/Macrophage (HLCZ01 + HBV) represents macrophages were cultured in 6-well plates, and hepatocytes (HLCZ01 cells and HLCZ01 + HBV cells) were cultured in 24 mm transwells with 0.4 µm pore polycarbonate membrane inserts. HLCZ01 (Macrophage)/HLCZ01 + HBV (Macrophage) represents hepatocytes (HLCZ01 cells and HLCZ01 + HBV cells) were cultured in 6-well plates, and macrophages were cultured in 24 mm transwells with 0.4 µm pore polycarbonate membrane inserts. (**B**) qPCR analysis of HBV DNA and ELISA analysis of HBsAg in Blank Medium, HLCZ01 cells supernatant and HLCZ01 + HBV cells supernatant with a molecular weight greater than 5 kDa or less than 5 kDa. UD, undetected. (**C**) qPCR analysis and western blot analysis of KDM7A in macrophage exposed or not to HBV for 48 h. After concentration by filtering device, the supernatant of HLCZ01 + HBV cells with a molecular weight greater than 5 kDa contained viral particles and proteins, while other components were absent. Experiments were independently repeated two or three times with similar results. The results are means and standard deviations. *, *P* ≤ 0.05; **, *P* ≤ 0.01; ***, and *P* ≤ 0.001 (versus control).

The difficulty of HBV clearance is mainly due to two reasons. On the one hand, the structure of cccDNA is considered far from stable and cannot be destroyed. On the other hand, the immune system of the host is unable to mount effective immune responses against HBV (3). As confirmed above, HBV infection upregulated the expression of KDM7A (Fig. 1B and C), and KDM7A can interact with cccDNA (Fig. 2I) correspondingly in human hepatocytes. Macrophages are a type of immune cell in the human immune system, and their internal environment is likely to be regulated by HBV (27). To verify whether the expression of KDM7A in macrophages is regulated by HBV, hepatocytes and macrophages were cocultured. We used 24 mm transwells with 0.4 µm pore polycarbonate membrane inserts and 6-well plates to coculture hepatocytes and macrophages. Macrophage (HLCZ01)/Macrophage (HLCZ01 + HBV) represents macrophages were cultured in 6-well plates, and hepatocytes (HLCZ01 cells and HLCZ01 + HBV cells) were cultured in 24 mm transwells with 0.4 µm pore polycarbonate membrane inserts. HLCZ01 (Macrophage)/HLCZ01 + HBV (Macrophage) represents hepatocytes (HLCZ01 cells and HLCZ01 + HBV cells) were cultured in 6-well plates. and macrophages were cultured in 24 mm transwells with 0.4 µm pore polycarbonate membrane inserts. The relative mRNA expression and protein level of KDM7A showed that HBV can upregulate the expression of KDM7A in macrophages (Fig. 4A). HBV is not only able to produce new viral particles and proteins with the help of host cells but also affects the biological processes of the host cells. To investigate which factor affects the expression of KDM7A in macrophages during coculturing, we collected different cell supernatants (un)infected with HBV for centrifugal concentration. Each sample was concentrated twofold using an Amicon Ultra-15 centrifuge filter unit with a 5-kDa molecular weight cutoff (EMD Millipore, USA). The cell supernatants of each group were divided into two fractions, greater than 5 kDa and less than 5 kDa, with or without viral particles and proteins, respectively. We quantified the HBV DNA by qPCR and deleted the expression by ELISA. As shown in Fig. 4B, HBV particles and proteins only existed in the supernatant that had greater than 5 kDa of HLCZ01 + HBV cells. Then they were added to macrophages separately, and the expression of KDM7A at the mRNA and protein levels was analyzed. Compared to the control group, the results showed that only the supernatant that had greater than 5 kDa of HLCZ01 + HBV cells can promote the expression of KDM7A (Fig. 4C). These data suggested that HBV may alter the immune microenvironment by increasing the expression of KDM7A to facilitate HBV persistence.

### The JAK-STAT signaling pathway activated by IFN-γ can be inhibited by KDM7A

**Fig 5:**
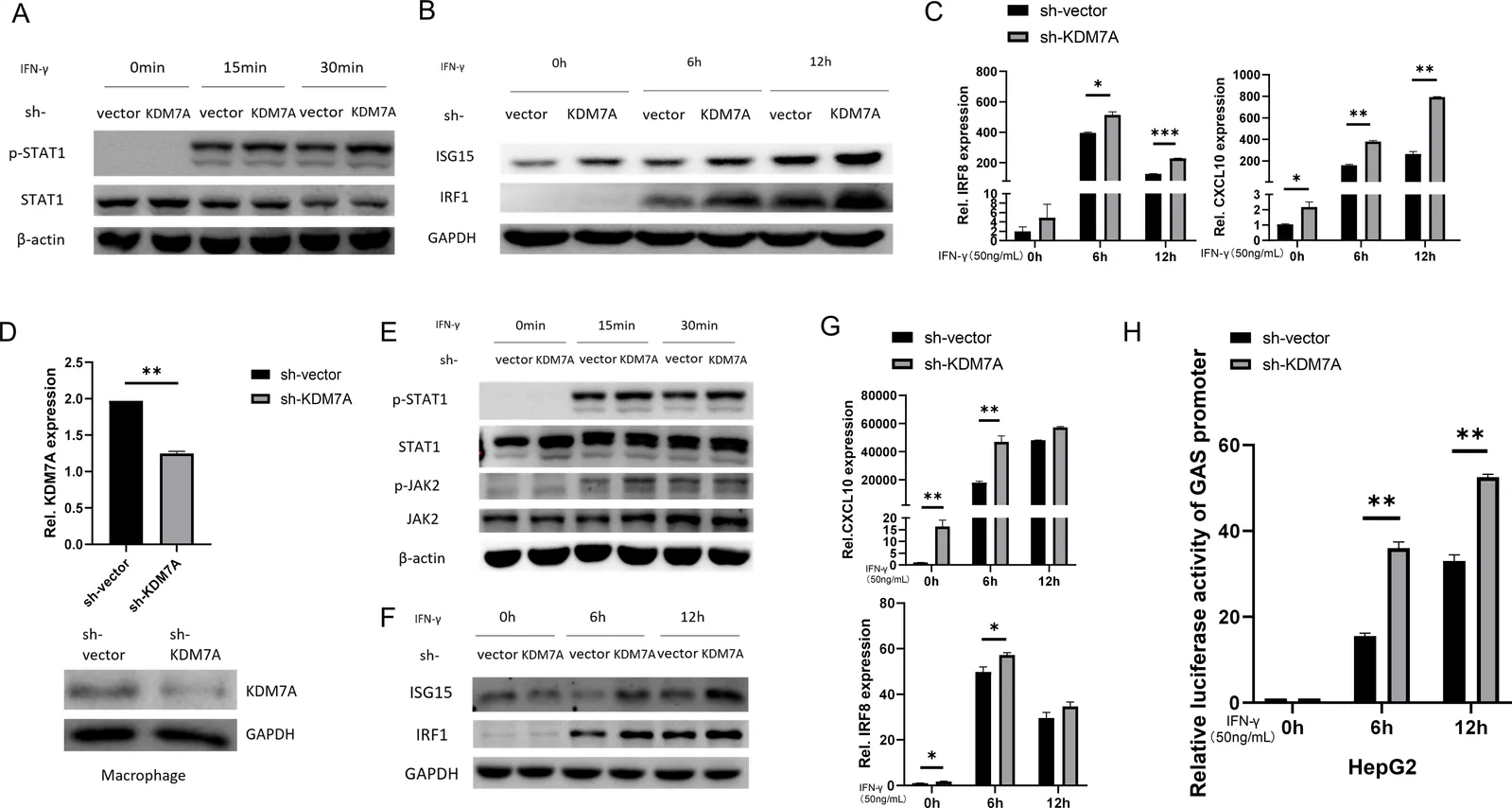
The JAK-STAT signaling pathway activated by IFN-γ is inhibited by KDM7A. HepG2-sh-vector and HepG2-sh-KDM7A cells were stimulated with IFN-γ (50 ng/mL). (**A**) Western blot analysis of STAT1 and STAT1 phosphorylation at 0 min, 15 min, and 30 min, (**B**) ISG15 and IRF1 at 0 h, 6 h, and 12 h after stimulation with IFN-γ were performed. (**C**) qPCR analysis of IRF8/CXCL10 at 0 h, 6 h, and 12 h after stimulation with IFN-γ were performed. (**D**) Macrophages were infected with lentivirus harboring sh-vector/KDM7A, respectively, for 72 h, qPCR analysis and western blot analysis of KDM7A in macrophages were performed. GAPDH was used as the internal control. (**E**) Macrophages were infected with lentivirus harboring sh-vector/KDM7A, respectively, for 72 h and then stimulated with IFN-γ (50 ng/mL). Western blot analysis of the expression of STAT1 and JAK2 and STAT1 and JAK2 phosphorylation in macrophages were performed at 0 min, 15 min, and 30 min after stimulation with IFN-γ was performed. Macrophages were infected with lentivirus harboring sh-vector/KDM7A, respectively, for 72 h and then stimulated with IFN-γ (50 ng/mL). (**F**) Western blot analysis of IRF1/ISG15 in macrophages and (**G**) qPCR analysis of IRF8/CXCL10 at 0 h, 6 h, and 12 h after stimulation with IFN-γ were performed. GAPDH was used as the internal control. (**H**) HepG2-sh-vector and HepG2-sh-KDM7A cells were transfected with pGAS-Luc. Relative luciferase activity of the GAS promoter was analyzed at 0 h, 6 h, and 12 h after IFN-γ (50 ng/mL) stimulation. GAPDH was used as the internal control. Experiments were independently repeated three or four times with similar results. The results are means and standard deviations. *, *P*＜0.05; **, *P*＜0.01; and ***, *P*＜0.001 (versus control).

Interferon is a key regulator of the immune response process, and it is also one of the first lines of defense for host cells against viruses (4). IFN-α is the only immunomodulator approved for HBV clinical therapy, and it can reduce HBV DNA, HBeAg, and HBsAg levels in hepatocytes (28, 29). However, instead of being widely used in the treatment of anti-HBV, type II interferon (IFN-γ) mainly participates in immune and inflammatory responses (6). In addition, IFNs serve as activators of the JAK-STAT signaling pathways which play important roles in many crucial biological processes, including cell proliferation, differentiation, and immune regulation (7). Moreover, as a member of the KDM family, KDM3A functions as an essential factor for the activation of the JAK2-STAT3 signaling pathway (17). We confirmed that HBV can upregulate the expression of KDM7A in hepatocytes (Fig. 1B and C) and macrophages (Fig. 4A). Combining previous studies and our experimental results, we next focused on the function of KDM7A in the IFN-γ/JAK2/STAT1 signaling pathway. IFN-γ can bind to its receptor, activate JAK1 and JAK2, and promote STAT1 dimerization. STAT1 binds a distinct DNA element named the GAS promoter and directly activates a distinct set of interferon-stimulated genes, notably ISG15, chemokines such as CXCL10, and transcription factors including IRF1 and IRF8 (29). We used HepG2-sh-vector and HepG2-sh-KDM7A cell lines, which were introduced before, and the expression of KDM7A was decreased (Fig. 2A). As shown in Fig. 5A, under IFN-γ stimulation, the phosphorylation of STAT1 in hepatocytes was enhanced by KDM7A knockdown. Subsequently, we tested the expression of ISG15 and IRF1 by western blotting (Fig. 5B) and the relative RNA expression of IRF8 and CXCL10 by qRT-PCR under the same stimulation (Fig. 5C). The results showed that ISG expression can be affected by KDM7A. As IFN-γ was originally identified as a “macrophage activating factor” and the expression of KDM7A can be upregulated in macrophages, we wondered if the JAK2-STAT1 signaling pathway could also be activated by KDM7A knockdown in macrophages. Macrophages were successfully infected with lentivirus harboring shRNA (Fig. 5D), and the phosphorylation of STAT1 and JAK2 was increased by the low expression of KDM7A in response to IFN-γ stimulation (Fig. 5E). The expression of ISGs (ISG15, IRF1, CXCL10, and IRF1) also showed the same trend (Fig. 5F and G). Next, we examined the activation of GAS luciferase triggered by IFN-γ, and the data showed that KDM7A knockdown increased GAS promoter activity (Fig. 5H). In summary, KDM7A inhibited the JAK-STAT signaling pathway activated by IFN-γ.

### KDM7A reduces the methylation of STAT1 and JAK2

**Fig 6:**
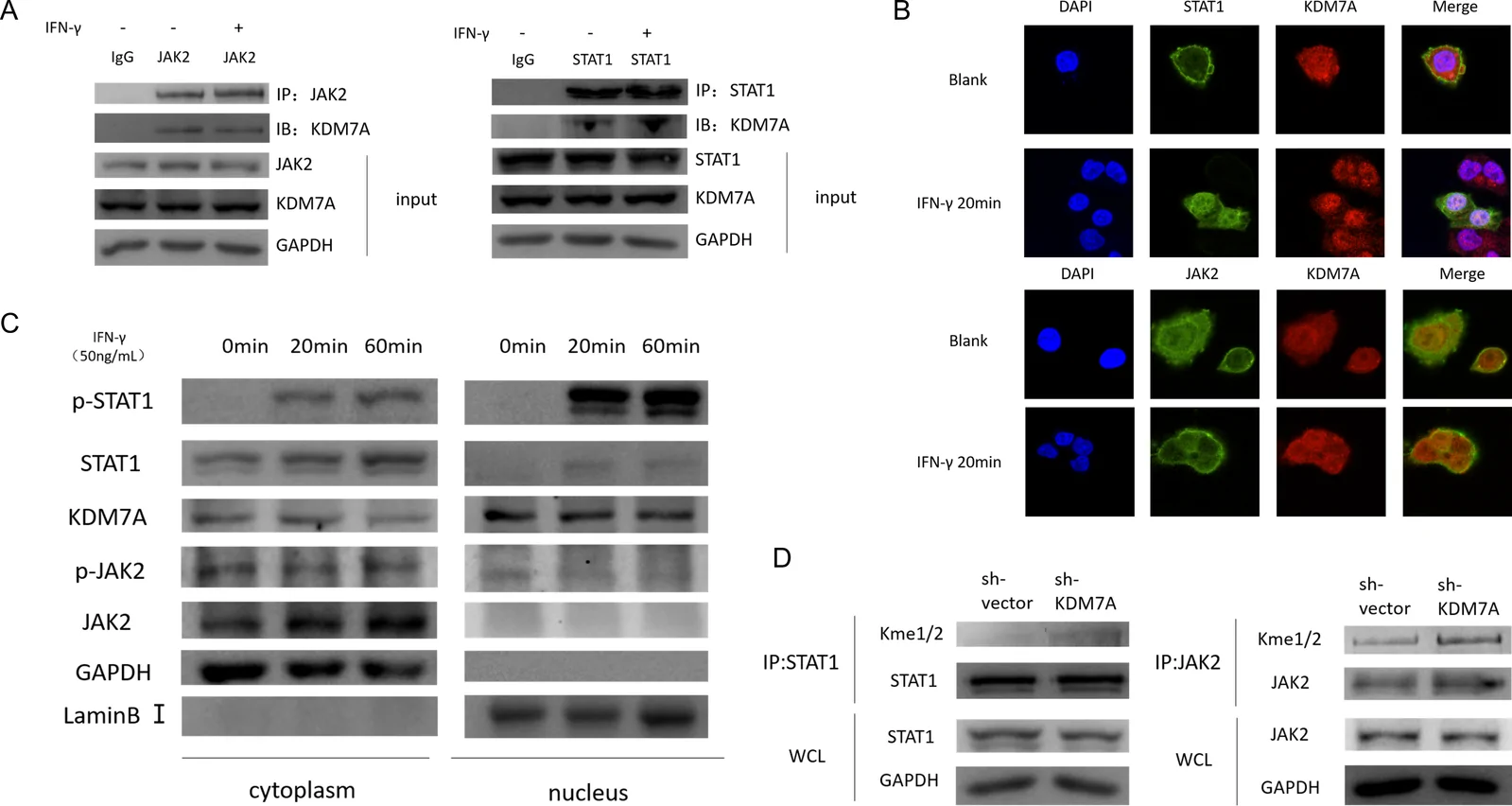
KDM7A inhibits the methylation of STAT1 and JAK2. (**A**) HepG2 cells were treated with IFN-γ (50 ng/mL）and were collected and subjected to immunoprecipitation (IP) assay. STAT1/JAK2 and KDM7A were detected by Co-IP and immunoblotting with the indicated antibodies. (**B**) The subcellular localization of KDM7A and JAK2/STAT1 in HepG2 cells was visualized using immunofluorescence with anti-KDM7A and anti-JAK2/STAT1 antibodies. The cells were treated with IFN-γ (50 ng/mL) for 20 min. Cell nuclei were counterstained by DAPI (blue). (**C**) HepG2 cells were treated with IFN-γ (50 ng/mL), and then the nucleus and cytoplasm of HepG2 cells were extracted at 0 min, 20 min, and 60 min. Western blot analysis of KDM7A, STAT1, and JAK2, and the phosphorylation of STAT1 and JAK2 were performed. (**D**) HepG2-sh-vector and HepG2-sh-KDM7A cells were collected and subjected to the IP assay. Kme1/2 of JAK2 and STAT1 were detected by Co-IP and immunoblotting with the indicated antibodies. Experiments were independently repeated three or four times with similar results.

Previous studies have shown that the expression of JAK2 and STAT1 is affected by methylation (30, 31). However, as a histone demethylase, the demethylation of non-histone proteins by KDM7A is poorly understood. To investigate the effect of KDM7A on STAT1 and JAK2 activity, we first tested the interaction between KDM7A and JAK2 or STAT1. The Co-IP assay confirmed the interaction of KDM7A with JAK2 or STAT1 (Fig. 6A). Additional immunofluorescence assays (Fig. 6B) and western blot assays of nuclear and cytoplasmic separation (Fig. 6C) of HepG2 cells were performed to confirm the colocation between KDM7A and JAK2 or STAT1. The colocation between KDM7A and STAT1 occurred mainly in the cytoplasm without IFN stimulation and occurred in the nucleus upon IFN stimulation. The colocation between KDM7A and JAK2 occurred in the cytoplasm of hepatocytes with or without interferon stimulation. We examined whether KDM7A, as a demethylase, can decrease the methylation of STAT1 and JAK2. Interestingly, KDM7A knockdown enhanced the mono-methylation and di-methylation of both JAK2 and STAT1 (Fig. 6D). It was demonstrated that KDM7A can interact with STAT1 and JAK2 while reducing their methylation.

## DISCUSSION

**Fig 7:**
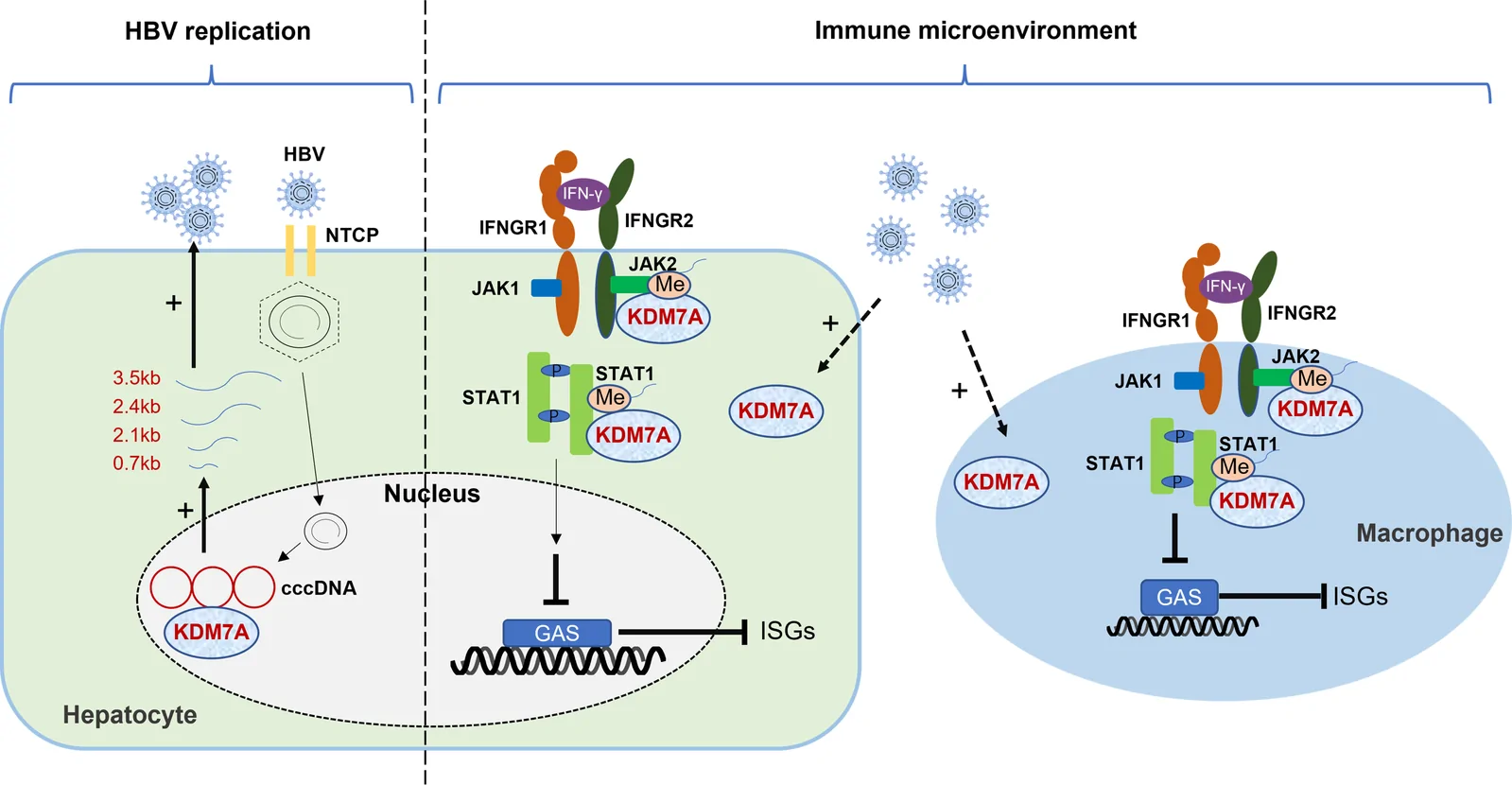
Schematic model of the dual functions of KDM7A in HBV replication and immunity system. On the left, KDM7A promotes the replication of HBV by interacting with cccDNA. On the right, the IFN-γ/JAK2/STAT1 signaling pathways in hepatocytes and macrophages are inhibited by KDM7A. Moreover, KDM7A inhibits the methylation of JAK2 and STAT1. Besides, KDM7A is highly expressed in HBV-infected cells.

In this study, we identified novel roles of KDM7A in HBV replication and the immune microenvironment through two subjects: the pathogen and host (Fig. 7). First, KDM7A is highly expressed in HBV-infected cells and promotes HBV replication *in vitro* and *in vivo*. Moreover, KDM7A augments its interaction with cccDNA and enhances HBV core promoter activity in hepatocytes. The specific mechanism needs to be elucidated. Because the mechanism of action of KDM7A and the transcription of cccDNA can be affected by increasing the methylation of cccDNA-involved histones (9, 19), we speculated that silencing KDM7A may increase H3K27 and H3K9 methylation levels on cccDNA-related histones to inhibit HBV replication. In Fig. 2H, the change in SP1 is different from that in Xp and Cp, and we speculated that there was another mechanism for the function of KDM7A on the HBV S promoter, but the details need further research. However, the activation of the S promoter is not contradictory to the downregulation of HBsAg. The reason is that the HBV core promoter plays an important role as it directs the initiation of transcription for the synthesis of pregenomic (pg) RNA (32). Pregenomic RNA contains all the genetic information that is necessary for subsequent HBV replication. pgRNA is the template for the reverse transcription of rcDNA, which is transcribed and translated to produce HBsAg. Therefore, the level of HBsAg can be affected by the activation of the HBV core promoter. In Fig. 2H, the effect of KDM7A on the HBV core promoter was more obvious than the effect of KDM7A on the HBV S promoter.

Second, we found that KDM7A can remodel the host immune environment. As a type of immune cell, macrophages can be affected by KDM7A. In the case of HBV infection, the expression of KDM7A in macrophages is significantly elevated. Besides, KDM7A can inhibit the JAK2-STAT1 signaling pathway in macrophages under the stimulation of IFN-γ. Interferons are key regulators of the immune response process against viruses. In the clinical treatment for HBV, type I IFN (IFN-α) plays a key role in the therapy for chronic HBV infection (33). However, the primary role of type II IFN (IFN-γ) is to regulate the immune system (6). In this article, our studies related to IFN-γ mainly focused on the regulation of internal macrophages by KDM7A in the presence of IFN-γ rather than on the antiviral effects of IFN-γ. In addition, macrophages can differentiate into two major subpopulations with different functions, including classically activated or inflammatory (M1) and alternatively activated or anti-inflammatory (M2) macrophages. IFN-γ can act as an activator of inflammatory (M1) macrophages (34), and the polarization of macrophages may be another factor that affected the immune microenvironment in our study.

Third, we found that KDM7A inhibited the production of ISGs through the IFN-γ/JAK2/STAT1 signaling pathway, and we tested the expression of partial ISGs such as ISG15, IRF1, IRF8, and CXCL10. In a previous study, ISGs were strongly correlated with the immune microenvironment. For example, serum CXCL10 is a peripheral biomarker that correlates with liver gene expression and differentiates immune-high and immune-low microenvironments. CXCL10 also correlated with intrahepatic immune cell signatures for T cells, B cells, and monocytes as well as the IFN-α and IFN-γ signaling pathways (35). In addition, as a histone demethylase, KDM7A may affect the expression of ISGs by directly influencing the post-translational modification process of ISG-related histones. Therefore, we hypothesized that KDM7A could remodel the immune microenvironment not only by affecting the internal environment of macrophages but also by influencing the expression of ISGs either directly or indirectly. Briefly, we identified novel roles of KDM7A in HBV replication and the immune microenvironment through two subjects: the pathogen and host. KDM7A is highly expressed in HBV-infected cells and promotes HBV replication *in vitro* and *in vivo*. Moreover, KDM7A interacts with HBV cccDNA and augments the activity of the HBV core promoter. On the other hand, KDM7A can remodel the immune microenvironment. It inhibits the expression of interferon-stimulated genes through the IFN-γ/JAK2/STAT1 signaling pathway in both hepatocytes and macrophages. Further study showed that KDM7A interacts with JAK2 and STAT1 and affects their methylation. In general, this study offers new viewpoints on the treatment of HBV and immunotherapy.

## MATERIALS AND METHODS

### Mice

C57BL/6 mice (6 weeks of age, male) were purchased from Nanjing GemPharmatech Co., Ltd. All animals were kept, and animal experiments were conducted in specific pathogen-free conditions. The experiments were conducted according to the institutional guidelines of Hunan University. To knock down the expression of Kdm7a in mice tissues, we purified lentivirus expressing sh-Kdm7a and sh-vector, respectively. We divided the mice into two groups. One group was injected with lentivirus-sh-vector (1 × 10^11^ PFU/g), and the other group was injected with lentivirus-sh-Kdm7a (1 × 10^11^ PFU/g). All the animals received the injection from the tail intravenously once every 3 d for three times. Three days after the lentivirus injection, the mice were given a hydrodynamic tail vein injection of pAAV/HBV1.2.

### Lentivirus production and purification for mouse injection

HEK293T cells were plated on 10 cm dishes. Lipofectamine 2000 (Thermo Fisher Scientific) was used to transfer the packaging vectors pSPAX2 (8 mg), envelope plasmid pMD2G (2.7 mg), and the targeted plasmid encoding shRNA (8 mg) on the next day. After 12 h, the cells were cultured with fresh medium. The supernatants were collected four times at 36 h, 48 h, 56 h, and 72 h after the transfection and clarified by filtration through 0.45 mm syringe filters. To knock down Kdm7a in mice, lentivirus-sh-vector and lentivirus-sh-Kdm7a were purified. The purification process of lentivirus was reported previously (36).

### Cell culture and reagents

The HLCZ01 cell line is a novel hepatoma cell line constructed in our laboratory. It supports the entire life cycle of HBV and HCV (20). HepG2 cells were purchased from ATCC (Manassas, VA, USA). HepAD38 cells gifted by Professor Guozhong Gong (Central South University) are capable of stably producing HBV. Macrophages (THP-1 cells) were obtained from ATCC. HEK293T cells were purchased from Boster. Huh7 cells were purchased from ATCC. The specific method of construction of HepG2-NTCP cells was described in previous literature (37). Briefly, HepG2 cells were infected with a lentivirus harboring an encoded human NTCP, and infected cells were selected with puromycin (2 ug/mL) to screen the best growing single cell clones for their ability to support HBV infection. The screening methods of HepG2-sh-vector/HepG2-sh-KDM7A cell lines are similar to HepG2-NTCP, the lentivirus harboring an encoded human NTCP was replaced by lentivirus harboring shRNA. HLCZ01 cells were cultured in collagen-coated tissue culture plates and cultured with DMEM/F12 medium supplemented with 10% (vol/vol) FBS (Gibco), 40 ng/mL of dexamethasone (Sigma), ITS (Lonza), penicillin, and streptomycin. HepG2 cells, HepAD38 cells, Huh7 cells, and HEK293T cells were propagated in Dulbecco’s modified Eagle medium (DMEM) supplemented with 10% FBS, L-glutamine, non-essential amino acid, penicillin, and streptomycin. The cell coculture of HLCZ01/HBV infected HLCZ01 and macrophages, macrophages were propagated in RPMI 1640 medium supplemented with 10% FBS and 0.05 mM β-mercaptoethanol. A 24 mm transwell with 0.4 µm pore polycarbonate membrane insert and sterile from Corning were used to coculture cells. The method of HBV infection with HLCZ01 follows the previous reports of our laboratory (20). Recombinant human IFN-γ (300-02) was purchased from PeproTech.

### Antibodies

Antibodies used include anti-KDM7A (Abclonal, A8266), anti-HBV core (Abcam, ab8637), anti-HBsAg (Novus biologicals, NB100-62652), anti-JAK2 (CST, 3230S), anti-p-JAK2 (CST, 3771S), anti-STAT1 (CST, 14995S), anti-p-STAT1 (CST, 9167S), anti-ISG15 (Abcam, 285367), anti-IRF1 (CST, 8478S), anti-Mono/Di Methyllysine (PTM BIO, PTM 602), anti-LMNB1 (CST, 17416S), and anti-GAPDH (Millipore, MAB374). Anti-β-actin was obtained from Sigma. The secondary antibody goat anti-rabbit IgG-horseradish peroxidase (HRP) was purchased from CST. Goat anti-mouse IgG-HRP secondary antibody was from Merck.

### HBV-infected cell culture

HBV was derived from the supernatant of HepAD38 cells capable of stably producing viral particles and was purified and concentrated by sucrose gradient ultracentrifugation. Cells were inoculated on collagen-coated plates infected with HBV (MOI of 200 genome equivalents per cell) in the presence of 4% polyethylene glycol (PEG) 6000 for 20–24 h. The inoculum was removed by washing liberally with PBS, and the cells were cultured in the presence of DMSO.

### Plasmid construction

The NTCP was constructed in pCDH-CMV-MCS-EF1 plasmid, the primers of NTCP were shown in Table 1. The short hairpin RNA targeting KDM7A was constructed in pSilencer-neo plasmid (Ambion). The target sequences of KDM7A shRNAs were shown in Table 1. The primers of HBV promoters to construct luciferase plasmids were listed in Table 1. pAAV/HBV1.2 was gifted by Professor Pei-Jer Chen (National Taiwan University). pGAS-luc (GAS luciferase plasmid) was obtained from Professor Zhenghong Yuan (Fudan University).

**TABLE 1:** Primers for the construction of plasmids

Primer	Sequence
NTCP(F)	5′- GGGGTACCATGGAGGCCCACAACGCGTCTG- 3′
NTCP(R)	5′- GCTCTAGAGGCTGTGCAAGGGGAGCAGTCCT- 3′
sh-RNA (target KDM7A)−1#	5′- CCCAAGCCATTTGTTCAGAAATATT- 3′
sh-RNA (target KDM7A)−2#	5′- CATCAGAAATTAGTCAGAGGGTACA- 3′
sh-RNA (target KDM7A)−3#	5′- TTAGACCTGGACACCTTATTA- 3′
HBV SpI(F)	5′- GGGGTACCTCGCAGACGAAGGTCTC- 3′
HBV SpI(R)	5′- CCGCTCGAGTGAGGCGCTATGTGTTG- 3′
HBV SpII(F)	5′- GGGGTACCCAATCCCAACAAGGAC- 3′
HBV SpII(R)	5′- CCGCTCGAGGGCCTGAGGATGAGTG- 3′
HBV core promoter(F)	5′- GGGGTACCTTGCCCAAGGTCTTAC- 3′
HBV core promoter(R)	5′- CCGCTCGAGGCTTGGAGGCTTGAAC- 3′
HBV X promoter(F)	5′- GGGGTACCAACCTTTTCGGCTCCT- 3′
HBV X promoter(R)	5′- CCGCTCGAGGCCATGGAAACGATGT- 3′

### Real-time PCR assay

The real-time PCR assay of mRNA refers to our previous report (38). The primers of qRT-PCR were shown in Table 2. For DNA detection, DNA was isolated from supernatant and mice serum. Real-time PCR for total HBV DNA was performed as described previously (39). The primers used for PCR to detect HBV DNA were 5′-CACCTCTGCCTAATCATC-3′(sense) and 5′-GGAAAGAAGT CAGAAGGCAA-3′(antisense). GAPDH was used as the internal control.

**TABLE 2:** Primers for qRT-PCR

Primer	Sequence
KDM7A(F)	5′- CCTTCACCCCACCAAGAGAC- 3′
KDM7A(R)	5′- AGACGTTGTTTGGCTGTTGC- 3′
Kdm7a-mouse(F)	5′- TGCAGCTCTACACGGCTCT- 3′
Kdm7a-mouse(R)	5′- CCAGCTTGAACAGGTTTGGAG- 3′
CXCL10(F)	5′- AGCAGAGGAACCTCCAGTCT- 3′
CXCL10(R)	5′- ATGCAGGTACAGCGTACAGT- 3′
IRF8(F)	5′- AGTAGCATGTATCCAGGACTGAT- 3′
IRF8(R)	5′- CACAGCGTAACCTCGTCTTC- 3′
HBV-3.5 kb RNA(F)	5′- CTCAATCTCGGGAATCTCAATGT- 3′
HBV-3.5 kb RNA(R)	5′- TGGATAAAACCTAGCAGGCATAAT- 3′
GAPDH(F)	5′- GAAGGTGAAGGTCGGAGTCA- 3′
GAPDH(R)	5′- AATGAAGGGGTCATTGATGG- 3′
GAPDH-mouse(F)	5′- CAGGAGAGTGTTTCCTCGTCC- 3′
GAPDH-mouse(R)	5′- TTCCCATTCTCGGCCTTGAC- 3′
cccDNA(F)	5′- CAAGACAGGTTTAAGGAGAC- 3′
cccDNA(R)	5′- GAGAGAAAGGCAAAGTGGAT- 3′

### Western blotting

The process of western blot refers to the previous report (40).

### Southern blotting

Wash the cells three times with PBS with lysate (50 M Tris-HCL, pH 8.0; 1 mm EDTA; NP40 0.2%; 150 mM NaCl), centrifuge, and remove nuclei. Add DNA digestion and pellet the supernatant with PEG 8000. Add proteinase K for digestion and precipitated pellets were lysed. The lysates were extracted with phenol and phenol:chloroform, followed by digestion with ethanol precipitation. DNA was isolated by 1% agarose electrophoresis, transferred to a nylon membrane, and molecularly hybridized with a DIG kit.

### ELISA

According to the product manual, HBsAg in culture supernatants was detected using commercial ELISA Kits (Shanghai Kehua Biological Engineering Co., KHB).

### Luciferase assay

Luciferase activity was measured using a dual-luciferase reporter assay system (Promega). Cell luciferase activity was normalized to the protein concentration measured by Bradford (41).

### Immunoprecipitation and chromatin immunoprecipitation

IP and ChIP procedures were published previously (42, 43). Specific primers used for qRT-PCR to determine the enrichment of cccDNA were listed in Table 2.

### Immunofluorescence

HepG2 cells were cultured in a 6-well plate for 24 h. Then the cells were fixed in 4% paraformaldehyde and washed three times with PBS. Cells were permeabilized with PBS containing 0.2% Triton X-100 for 15 min and blocked with goat serum (diluted to 1:50 in PBS) for 1 h at room temperature. Cells were then incubated overnight at 4°C with anti-STAT1/JAK2 and anti-KDM7A, followed by incubation with fluorescent (FITC)-labeled goat anti-rabbit IgG or goat anti-mouse IgG for 1 h. The nuclei of the cells were counterstained with DAPI. Fluorescent images were observed under the fluorescent microscope (Olympus).

### Nuclear and cytoplasmic extraction

The nuclear and cytoplasmic extraction were published previously (40).

### Statistical analysis

All experiments were replicated at least two times, and the data from parallel cultures were acquired. The data are presented as means ± SD. The data were analyzed using a two-tailed Student’s *t*-test. *P < 0.05, **P < 0.01, ***P < 0.001.
